# Arctic amplification modulated by Atlantic Multidecadal Oscillation and greenhouse forcing on multidecadal to century scales

**DOI:** 10.1038/s41467-022-29523-x

**Published:** 2022-04-06

**Authors:** Miao Fang, Xin Li, Hans W. Chen, Deliang Chen

**Affiliations:** 1 Northwest Institute of Eco-Environment and Resources, Chinese Academy of Sciences, Lanzhou, China; 2 National Tibetan Plateau Data Center, State Key Laboratory of Tibetan Plateau Earth System, Environment and Resources (TPESER), Institute of Tibetan Plateau Research, Chinese Academy of Sciences, Beijing, China; 3 CAS Center for Excellence in Tibetan Plateau Earth Sciences, Chinese Academy of Sciences, Beijing, China; 4 Department of Physical Geography and Ecosystem Science, Lund University, Lund, Sweden; 5 Regional Climate Group, Department of Earth Sciences, University of Gothenburg, Gothenburg, Sweden

**Keywords:** Climate change, Palaeoclimate

## Abstract

Enhanced warming in the Arctic (Arctic amplification, AA) in the last decades has been linked to several factors including sea ice and the Atlantic Multidecadal Oscillation (AMO). However, how these factors contributed to AA variations in a long-term perspective remains unclear. By reconstructing a millennial AA index combining climate model simulations with recently available proxy data, this work determines the important influences of the AMO and anthropogenic greenhouse gas forcing on AA variations in the last millennium, leading to identification of a significant downward trend of AA on top of a sustained strong AMO modulation at the multidecadal scales. The decreased AA during the industrial era was strongly associated with the anthropogenic forcing, proving the emerging role of the forcing in reducing the AA strength.

## Abstract (web summary)

Reconstructed Arctic amplification shows a significant downward trend over the past millennium which can to a large part be explained by the strength of the Atlantic Multidecadal Oscillation and recent anthropogenic greenhouse gas forcing.

## Introduction

The Arctic has warmed more than twice the global average since the preindustrial era^1,2^, a phenomenon known as Arctic amplification (AA). This amplified warming has been associated with tremendous changes in the Arctic climate system, including shrinking sea-ice thickness and extent^3,4^, melting of the Greenland ice sheet^5^, increasing greenhouse gas emissions from thawing permafrost^6^, and disturbances to local ecosystems^7^. Many studies have linked AA to shifting weather patterns and extreme weather events in the mid-latitudes^8,9^, although this issue remains controversial^10^. Nevertheless, it is generally accepted that the rapid Arctic climate changes have had and will have far-reaching and profound impacts on human and natural systems^3^. Understanding how and why AA has changed over the last millennium provides a useful long-term perspective for the ongoing and future climate changes.

AA is evident in instrumental observations^2^ and climate model simulations^3^ and is commonly thought to be caused by positive ice-albedo feedback associated with sea-ice loss^2,4^. However, AA also occurs in models without ice-albedo feedback^11^, challenging the view that sea ice is the key factor for AA. So far, AA has been linked to additional local processes such as temperature, vegetation, water vapor and cloud feedback mechanisms^11–14^, and remote processes including changes in the atmospheric and oceanic heat transport to the Arctic^1,15,16^. Much uncertainty remains about the mechanisms behind and their relative importance to AA, as evidenced by the disagreement about future AA by model projections^17^ and the general overestimation of AA by models^18^. A better understanding of AA is crucial for reliable assessments of future Arctic and global climate change.

Limited by the length of instrumental observations, most studies on AA have focused on the industrial era characterized by anthropogenic warming^17–19^, especially during the last decades, which makes it difficult to distinguish natural and human-induced contributions to AA. Nevertheless, instrumental records indicate that AA has varied on multidecadal time scales during the recent century and co-varied with the phase of the Atlantic Multidecadal Oscillation (AMO)^1,19^. The observations furthermore suggest that AA was stronger during the early twentieth-century warming than the recent warming up till 2014^19^, indicating that the anthropogenic greenhouse gas forcing may have weakened AA. Placing these observations in a long-term perspective would allow for more robust assessments about the relative roles played by the AMO and anthropogenic forcing in modulating AA and an improved understanding of how AA was influenced by different factors under various climate states.

**Fig. 1:**
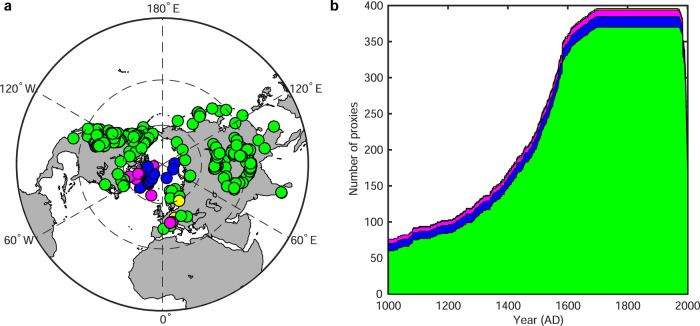
Spatial and temporal distributions of multiple types of proxy data used in this study. **a** Spatial distribution of the 396 temperature-sensitive proxy data of different types. **b** Number of proxies over time used in our paleoclimate data assimilation-based temperature reconstruction. Green: tree ring proxy; Blue: ice core proxy; Magenta: lake sediment proxy; Yellow: historical document proxy.

The AA signal can be detected in paleoclimate records^20^, but proxy-based reconstructions are typically too uncertain to reliably characterize AA on multidecadal time scales due to the sparseness of proxy data. To overcome this limitation, we reconstructed Northern Hemispheric (NH) annual near-surface temperature during the past millennium with a 2° spatial resolution using the paleoclimate data assimilation (PDA) approach^21^. The PDA approach is a state-of-the-art and best-of-both-worlds method for estimating past climate fields by assimilating proxy records into a climate model, and shows several distinct advantages over the existing climate field reconstruction methods^22–24^. We used the PDA method to assimilate 396 recently released, multi-type, annually resolved and temperature-sensitive proxy records (see Fig. 1 and Methods) from the PAGES2K Consortium^25^. The number and types of assimilated proxy records in this study, especially those over high latitudes, far exceed those in previous NH temperature reconstructions, creating a great potential to provide a more reliable estimate of AA over the last millennium than previous studies.

## Results and Discussion

Verification of the PDA-based temperature reconstruction (see Supplementary information) shows that the reconstruction agrees well with several observational temperature datasets during the instrumental period, and has a similar level of reliability as the Twentieth Century Reanalysis^26^ which assimilates surface pressure observations (see Fig. S1). In addition, the PDA-based reconstruction shows a high level of agreement with previous proxy-based reconstructions (average correlation of annual mean NH temperatures is *r* = 0.61, *p* < 0.001) (see Fig. S2). To further verify the reconstruction over the whole millennium, we conducted an experiment where we randomly selected 75% of the proxy records to use in the assimilation, and withheld the remaining 25% for verification. This proxy-based verification confirms that the temperature estimates in the PDA-based reconstruction agree more closely with the withheld proxies than the unconstrained model run (see Fig. S3).

**Fig. 2:**
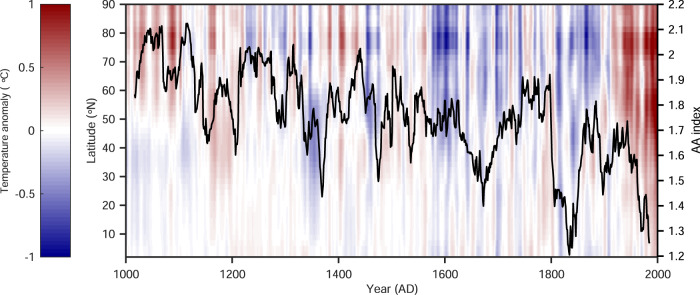
Annual-mean zonal variability of the temperature anomalies over the NH during the past millennium and the reconstructed AA index. The black line is the reconstructed AA index derived from the PDA-based reconstruction.

AA is typically defined as the ratio between a change in Arctic temperature to a corresponding change in NH or global temperature over a specific period. Here we use a robust metric based on the slope of a linear regression between Arctic (northward of 60°N) and NH annual temperature anomalies over a 31 years moving-window period (see Methods), which has been shown to possess favorable statistical properties, especially when the NH temperature change is small^27,28^. Based on the millennial PDA-based temperature reconstruction during 1000 and 2000, we obtained centered AA index values for 1015–1985 with a sliding window of 31 years. Because AA estimates for the sliding window are placed at the middle of the window, there are no estimates for the first and the last 15 years. Note that the trend in millennial AA indices derived using this method is not sensitive to the selection of moving-window period (see Fig. S4). Figure 2 shows the annual-mean zonally averaged temperature anomalies from the PDA-based temperature reconstruction and the corresponding derived AA index. Over the past millennium, the AA index ranged from 1.21 to 2.12 with a mean of 1.76, indicating that the amplification of Arctic temperature changes (AA index > 1) is an inherent feature of the climate system regardless of warm or cold periods, which is consistent with the findings of previous paleoclimate studies^20^. The reconstructed AA index exhibits strong multidecadal variations and was strongest during the Medieval Climate Anomaly (1015–1100). In contrast, AA was relatively weak during the industrial era (1850–1985), with a mean AA index of 1.58 as compared with 1.65 during the preindustrial era (1714–1849).

**Fig. 3:**
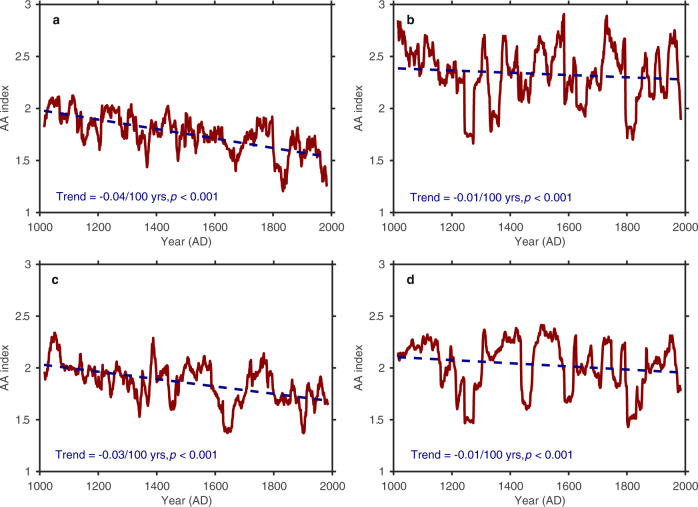
The reconstructed AA indices and estimated AA trends during the past millennia derived from three PDA-based reconstructions and the mean of CMIP5 multi-model simulations. **a** PDA-based reconstruction of this study. **b** Goosse2012. **c** Steiger2018. **d** Multi-model mean. The dark red solid line represents the AA index derived from PDA-based reconstructions or the mean of CMIP5 multi-model simulations, and the dark blue dotted line is the long-term trend. The AA index values were computed based on annual temperature anomalies without smoothing with respect to the mean for 1961–1990. The long-term trends were computed via Mann-Kendall trend detection (see Methods) for each AA index time series.

Over the past millennium, the reconstructed AA index exhibits a statistically significant (95% confidence level) downward trend of −0.04/100 years (*p* < 0.001, see Fig. 3a). Significant negative AA index trends are also found in a previous PDA-based reconstruction^22^ (hereinafter Goosse2012) (−0.01/100 years, *p* = 0.008, see Fig. 3b) and a new global hydroclimate and dynamical variables reconstruction over the Common Era using data assimilation^29^ (hereinafter Steiger2018) (−0.03/100 yrs, *p* < 0.001, see Fig. 3c), suggesting consistencies among the three PDA-based reconstructions on reflecting the AA variation trends, though there are differences in the strengths of the trends and the magnitudes of the AA variations. These disagreements are not surprising, considering that the three PDA-based reconstructions used different prior ensembles from various climate models, data assimilation algorithms, ensemble sizes, proxy data and proxy system models (see details in Table S1). AA indices calculated from unconstrained model simulations from the Last Millennium and Historical experiments from phase 5 of the Coupled Model Intercomparison Project (CMIP5)^30^ are generally of similar magnitude but larger than the reconstructed AA index from this study and the Steiger2018 reconstruction and smaller than the AA index derived from Goosse2012 (see Fig. 3b). Additionally, most model simulations display a negative trend in the AA index (see Fig. S5), with a statistically significant (95% confidence level) negative trend in the multi-model mean of −0.01/100 yrs (*p* < 0.001) (see Fig. 3d). Given that averaging over model runs removes part of the internal variability, the declining AA index trend in the CMIP5 multi-model mean suggests that the weakening AA is a robust signal which may have been partly modulated by external forcing.

**Fig. 4:**
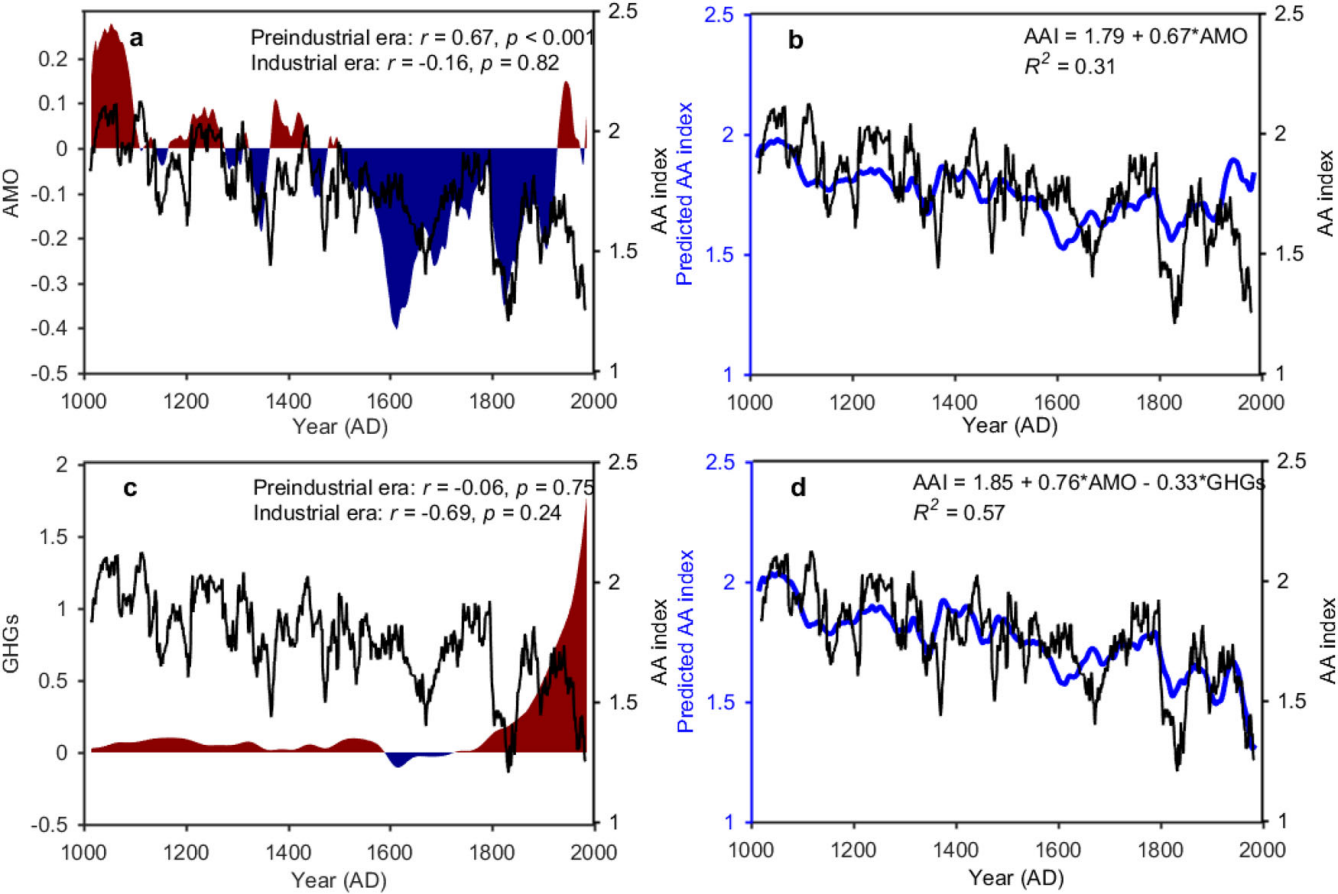
Co-variations between the reconstructed millennial AA index and GHGs forcing and AMO modes. **a** The reconstructed millennial AA index (black line) derived from the PDA-based reconstruction and the reconstructed AMO index (shading) based on multi-type proxies^33^. **b** Actual AA index (black line) and predicted AA index (blue line) based on multivariate linear regressions on only the AMO index. **c** The reconstructed millennial AA index derived from the PDA-based reconstruction and the reconstructed GHGs forcing (shading) used in the PMIP3 last-millennium project^35^. **d** Actual AA index (black line) and predicted AA index (blue line) based on multivariate linear regressions on both the AMO index and GHGs forcing. The AMO index and GHGs forcing have been smoothed using 30 year rolling means.

To investigate the roles played by key climatic indicators in the Arctic, large-scale internal modes of variability and different forcings on AA over the past millennium, we performed a correlation analysis between the millennial AA index and several reconstructed climate indices for local climate, important modes of climate variability as well as forcings, including Arctic sea-ice extent^31^, the phase of the El Niño/Southern Oscillation^32^, AMO^33^, Pacific Decadal Oscillation (PDO)^33^ and North Atlantic Oscillation (NAO)^34^, as well as radiative forcing from solar activity, well-mixed greenhouse gases (GHGs) and volcanic aerosols^35^. Although Arctic sea-ice loss is often thought to be the driving factor behind AA variations^2,4^, the correlation between AA variations and reconstructed sea-ice extent over the past millennium is weak and insignificant (*r* = 0.02, *p* = 0.91). However, because both negative and positive sea ice anomalies may amplify the temperature change in the Arctic during warming and cooling periods, respectively, a weak correlation between AAI and sea ice extend here may not necessarily mean that sea ice is not a driving factor behind AA variations at other time scales^2,4^. In addition, the low variability in the reconstructed Arctic sea ice extent series^34^ may also be a possible reason responsible for the weak correlation (see Fig. S6). The reconstructed AA index co-varied most closely with the phase of the AMO (Fig. 4a) on multidecadal time scales, with a significant (95% confidence level) positive correlation of *r* = 0.56 over the whole millennium (*p* = 0.001). The PDO shows a similar co-variability with the AA index as the AMO (see Fig. S7) but does not explain a significant portion of the remaining variance; thus, we focus on the AMO here.

The correlation between the phase of the AMO and the AA index is stronger during the preindustrial era (i.e., before 1850, hereinafter) (*r* = 0.67, *p* < 0.001) and is insignificant at the 95% confidence level during the industrial era (i.e., after 1850, hereinafter) (*r* = −0.16, *p* = 0.82). A proportion of the correlation between the AA and AMO index can be attributed to the common negative trend in both indices. After removing the linear trends in both time series, the correlation is decreased to *r* = 0.32 (*p* = 0.10) over the preindustrial era. Our results strengthen the conclusion from previous observation-based studies^1,19^ that the AMO has played an important role in modulating the strength of AA, and further extend that conclusion to multidecadal to century time scales in the past millennium.

The AMO alone cannot explain the decline in the AA index during the most recent century. In fact, the transition to a positive phase of the AMO before the turn of the 21st century should have favored a stronger AA, as shown by the predicted AA index in Fig. 4b based on a linear regression between the AA and AMO index. Out of the remaining factors considered in this study, radiative forcing from GHGs explains most of the remaining variance of the AA index. Although the correlation between the AA index and GHGs is weak and insignificant during the preindustrial era (*r* = −0.06, *p* = 0.75) because the concentration of GHGs was mostly stable during this time period, the correlation became strong during the industrial period (*r* = −0.69, *p* = 0.24), see Fig. 4c. Including both the AMO and GHGs as predictors for the AA index increases the explained variance from 31 to 57% compared to considering the AMO alone (Fig. 4d) and captures well the multidecadal variations and declining trend in the AA index during the industrial era. Furthermore, when considering both factors, the AMO shows a consistent modulation of the AA during both the preindustrial (partial correlation *r*_*p*_ = 0.70) and industrial (partial correlation *r*_*p*_ = 0.65) eras. This result suggests that GHGs may have played a significant role in weakening AA in the industrial era.

**Fig. 5:**
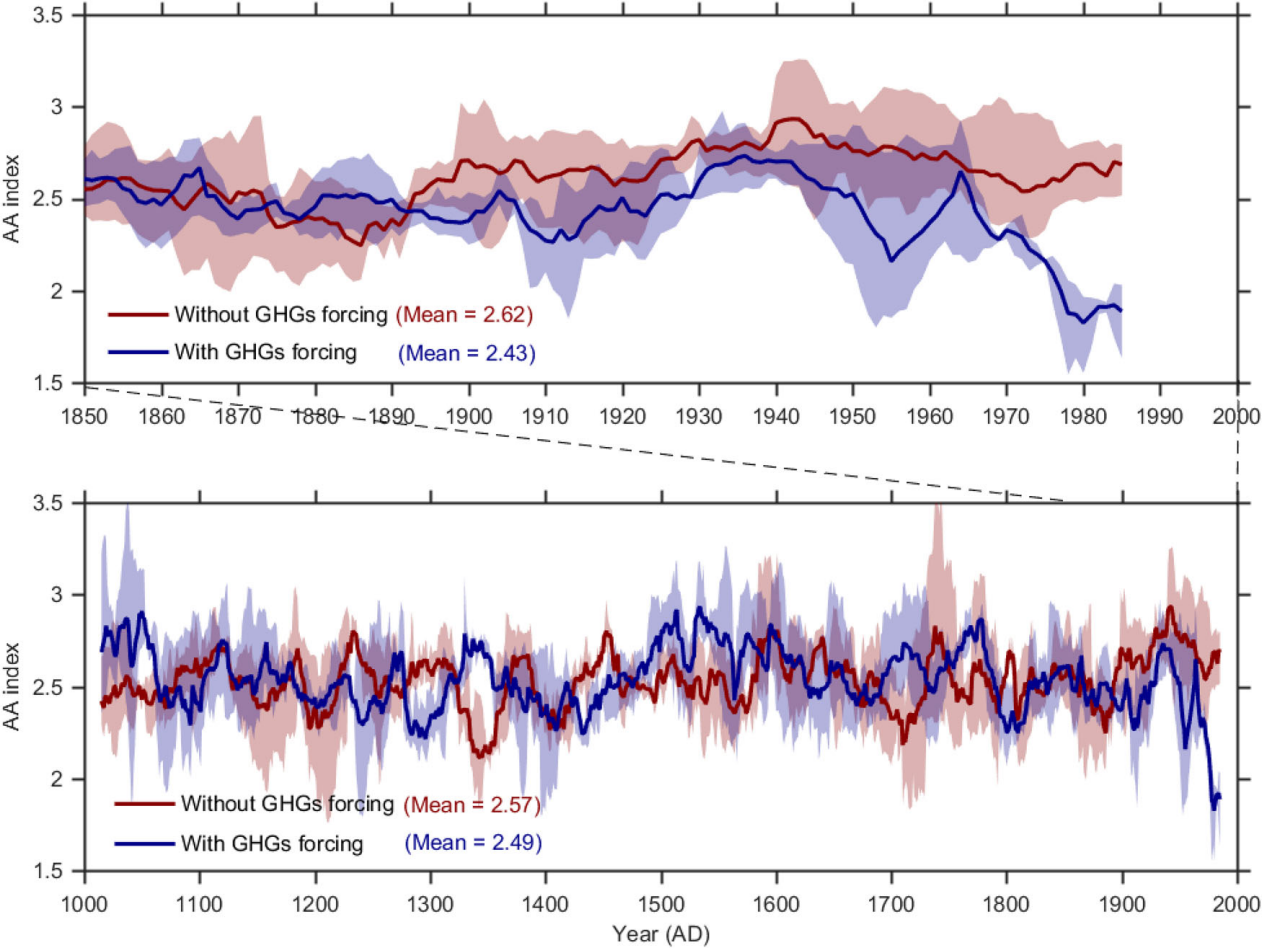
Variations of the AA indices without GHGs forcing and with GHGs forcing during the past millennium and the industrial era. The dark red line represents the ensemble mean of a three-member ensemble of climate model simulations driven with orbital forcing only. The dark blue line represents the ensemble mean of a three-member ensemble of climate model simulations driven with both orbital forcing and GHGs forcing. Light red and light blue shadings are minimum and maximum of the ensemble. The model simulations were provided by long-term ensemble simulation experiments^38^.

There is a solid theoretical basis that GHG forcing weakens AA because the GHG radiative forcing is strongest over the tropics^36^, although an analysis of model simulations has suggested that the negative effect of GHG on AA is small^37^. To conclusively determine whether GHGs have significantly contributed to the declining AA in the past century, we analyzed two millennium-scale model experiments running with and without GHG forcing^38^. Figure 5 shows the AA indices derived from these two model experiments. The experiment with GHG forcing shows a pronounced decline in the AA index after around 1900 which becomes clearly distinguished from internal variability around 1970. Hence, both the PDA-based reconstruction and controlled model experiments agree that the radiative forcing from GHGs has played an increasingly important role in reducing AA in the last century.

The modulation of AA by the AMO conforms with the observation that a large portion of regional and hemispheric NH temperature changes can be attributed to changes in North Atlantic sea surface temperature^39–43^. The AMO signal propagates throughout the NH via a sequence of atmospheric and oceanic teleconnections^40^ and partly modulates poleward energy transport via atmospheric and oceanic circulations^44–48^. In response to the strengthening AMO, the North Atlantic ocean fluxes release more latent and sensible heat into the atmosphere, strengthening sea-atmosphere interactions and poleward ocean and atmospheric heat transport, and vice versa during a weakening AMO^43,49–51^. Thus, we suggest that the AMO modulates AA on multidecadal to century time scales by altering the poleward ocean and indirectly the atmospheric heat transport of the NH. Our finding that the strength of AA is closely linked to the AMO on multidecadal time scales highlights the importance of the background internal climate variability related to the ocean circulation in regulating heat transport in the NH.

Here we have used a combination of paleoclimate proxy data and climate models to show that AA has exhibited a strong variability over a range of time scales during the past millennium. The reconstructed AA index reveals that near-surface temperature changes are invariably enhanced in the Arctic relative to the whole NH, suggesting that AA is an inherent feature of the global climate system driven by positive feedback mechanisms. Additionally, the strength of AA is strongly dependent on the phase of the AMO, with positive AMO phases favoring stronger AA. The fact that AA is linked to the AMO indicates that there is some predictability in AA on multidecadal time scales. This finding can partly explain why the spread in future model projections of AA correlates with the modeled position of the Intertropical Convergence Zone (ITCZ)^17^, given that the position of the ITCZ depends on the phase of the AMO^43^, and points to the possibility of using the AMO as an emergent constraint to reduce the uncertainty in projected future AA. Another important finding is that GHGs forcing has likely had a significant role in the apparent weakening AA during the 20^th^ century by heating near-surface temperatures more in the lower latitudes than the higher latitudes, resulting in lower AA values despite the enhanced Arctic warming and sea ice loss in recent decades. It is possible that the recent unparalleled global warming caused by anthropogenic activities will trigger feedback mechanisms that are unprecedented in the last millennium, which could have further enhanced AA in the recent decades. In fact, multiple lines of evidences show that the fast sea ice decline in the past decades has been unprecedented over the past 1,450 years^31^. This work provides a crucial baseline against which the ongoing and future changes in AA can be compared.

## Methods

### Paleoclimate data assimilation

An “offline” data assimilation category (or “no cycle” data assimilation) is employed to conduct our PDA experiment, where background ensembles were constructed from existing climate model simulations, and this background ensemble was used over the entire PDA process^23,52^. A time-averaged ensemble square root filter (EnSRF)^52^ was employed in our PDA experiment. Before presenting the details of the EnSRF, we first note that **X**^b^ is the prior estimate of the state vector (the so-called background, e.g., air temperature simulated by a climate model; in this study, the prior fields consist of an ensemble of annual mean values, randomly drawn from a long climate simulation), and **X**^a^ is the posterior state vector (i.e., the updated estimate of air temperature). Observations (e.g., proxies in this context) are contained in the vector **Y**°. The key equations of this approach are as follows.

1 $${{{{{{\bf{X}}}}}}}^{{{{{{\rm{a}}}}}}}={{{{{{\bf{X}}}}}}}^{{{{{{\rm{b}}}}}}}+{{{{{\bf{K}}}}}}\left[{{{{{{\bf{Y}}}}}}}^{{{{{{\rm{o}}}}}}}-{{{{{\rm{H}}}}}}\left({{{{{{\bf{X}}}}}}}^{{{{{{\rm{b}}}}}}}\right)\right]$$

2 $${{{{{{\bf{P}}}}}}}^{{{{{{\rm{a}}}}}}}=\left({{{{{\bf{I}}}}}}-{{{{{\bf{KH}}}}}}\right){{{{{{\bf{P}}}}}}}^{{{{{{\rm{b}}}}}}}$$

3 $${{{{{\bf{K}}}}}}={{{{{{\bf{P}}}}}}}^{{{{{{\rm{b}}}}}}}{{{{{{\bf{H}}}}}}}^{{{{{{\rm{T}}}}}}}{\left({{{{{{\bf{HP}}}}}}}^{{{{{{\rm{b}}}}}}}{{{{{{\bf{H}}}}}}}^{{{{{{\rm{T}}}}}}}+{{{{{\bf{R}}}}}}\right)}^{-1}$$

4 $${{{{{{\bf{P}}}}}}}^{{{{{{\rm{b}}}}}}}=\frac{1}{N-1}\mathop{\sum }\limits_{i=1}^{N}\left({{{{{{\bf{X}}}}}}}_{i}^{{{{{{\rm{b}}}}}}}-\left\langle {{{{{{\bf{X}}}}}}}^{{{{{{\rm{b}}}}}}}\right\rangle \right){\left({{{{{{\bf{X}}}}}}}_{i}^{{{{{{\rm{b}}}}}}}-\left\langle {{{{{{\bf{X}}}}}}}^{{{{{{\rm{b}}}}}}}\right\rangle \right)}^{{{{{{\bf{T}}}}}}}$$

where **P**^b^ is the error covariance matrix for the prior, **P**^a^ is the error covariance matrix for the posterior, **R** is the error covariance matrix for the observations, *N* is the ensemble size, and **I** is the identity matrix. 〈•〉 denotes the expectation of the ensemble. The estimated observations were obtained through H(**X**^b^), i.e., **Y**^e^ = H(**X**^b^), and H(•) is the observation operator that maps **X**^b^ from the state space to the observation space. The difference between the observations and the estimated observations, i.e., **Y**°–H(**X**^b^), is called the innovation. The Kalman gain **K** determines the distribution modes of observational information in space and time. The information contained in **K** allows for information from measurement locations to be spread to non-measurement locations in model space. **K** also acts as a weighting term that scales the innovation term (i.e., **Y**°–H(**X**^b^)) according to prior and observation errors.

### Proxy dataset and proxy forward model

The PAGES2K Consortium recently published a new-generation global proxy dataset, and the proxies included in this new-generation dataset have been ascertained to regionally covary with temperature^25^; hence, those proxies are well suited to reconstruct temperature. Because our aim is to reconstruct the temperature field with an annual time resolution, only proxies with an annual time resolution were considered in this study. Here we selected 396 proxies (see Fig. 1), including 370 tree ring proxies (width, density and mixed latewood density), 15 ice core proxies (δ^18^O), 9 lake sediment proxies (δ^18^O, varve thickness) and 2 historical documents (temperature series) from this new-generation proxy dataset. In addition, as shown in Eq. (1), the observed values of the proxy (i.e., **Y**°) are directly compared with the simulated values of the proxy (i.e., **Y**^e^) in the PDA algorithm to obtain the innovation. This comparison requires mapping of the climate state variables (e.g., air temperature) to the quantity that forms the proxy record (e.g., tree ring width). In this study, we used a linear-univariate proxy system model (PSM) (see Eq. (5)), which maps temperature to proxy measurements by fitting proxy data to gridded instrumental temperature data. Then, the linear-univariate PSM was used to predict proxy values from the prior estimate (i.e., **Y**^e^ = H(**X**^b^)). The effectiveness of the linear-univariate PSM in PDA has already been tested in previous cases^23,52,53^.

5 $${{{{{\bf{Y}}}}}}={\beta }_{0}+{\beta }_{1}* {{{{{\bf{T}}}}}}+\varepsilon$$

where vector **Y** denotes proxy values, vector **T** is the annual mean temperature anomalies, *β*_0_ and *β*_1_ are the intercept and slope, respectively, *ε* is a Gaussian random variable with zero mean and variance σ^2^, and σ^2^ is acquired during the calibration process and then used to define the diagonal elements of matrix **R** in Eq. (3). In our study, the calibration period was set to 1880–2000 AD. Gridded Goddard Institute for Space Studies (GISS) surface temperature analysis (GISTEMP temperature anomalies)^54^ was used to calibrate the PSM. The ordinary linear least squares approach was used to estimate the intercept *β*_0_, slope *β*_1_ and variance σ^2^. All 396 proxy data were assimilated without prefiltering the proxies to select those having a significant correlation with air temperature because the proxies with a large residual variance σ^2^ will have reduced weight in the updating process according to the principle of data assimilation^23,52^.

### Experimental designs

A 500-member static prior ensemble was randomly drawn from the MPI-ESM-P last millennium simulations^55^ from the “Coupled Model Intercomparison Project Phase 5 (CMIP5)”^30^ covering the period from 850–1850 CE (only 2 m air temperature was used). Before obtaining those 500-member ensemble samples, monthly MPI-ESM-P simulations were averaged to a calendar year, and the temporal mean over the entire dataset was removed. The resulting annual mean anomaly fields were then spatially interpolated to a 2° uniform grid. The 500-member ensemble sample was used for every year^23,52^. Notably, because our ensemble size is rather large with 500 members, we did not need to employ covariance localization, which is a common method of reducing the effects of sampling error in small ensembles^23,52^. Our PDA experiment was conducted to reconstruct 2 m air temperature fields during the period from 1000–2000 AD. In the PDA experiment, the assimilation was performed one year at a time, yielding an annual mean ensemble mean analysis for each year, which is the climate field reconstruction for that year. The reconstruction was repeated 50 times (each one being a “realization”) in a Monte Carlo fashion^23^, and each realization assimilated different observations obtained through randomly sampling 75% of the proxies. The mean of the 25,000 reconstruction realizations (50 Monte Carlo samples each having a 500-member ensemble) was taken as the analysis. This procedure resulted in a reconstructed 2 m air temperature field with an annual resolution at 2° spatial resolution.

### Definition of Arctic amplification index

The AAI is commonly defined as the ratio of some change in Arctic temperature to a corresponding change in the NH or globally. However, the use of this ratio as a measure of AA may result in some shortcomings, e.g., extreme values may occur when the denominator is close to zero^28,56^, and the use of the ratio may therefore lead to unrealistic estimates of AA^27^. This study employs a new measure of the AA^27^ to better characterize AA on a millennial time scale and avoid extreme values. This new measure of AA that links pan-Arctic (60°N–90°N) and NH (0°–90°N) temperature anomalies via a regression relationship is shown as follows:

6 $${{{{{{\bf{T}}}}}}}_{{{{{{\bf{Arc}}}}}}}={a}_{0}+{a}_{1}* {{{{{{\bf{T}}}}}}}_{{{{{{\bf{NH}}}}}}}+\varepsilon$$

where **T**_**Arc**_ represents the temperature anomalies over the pan-Arctic and **T**_**NH**_ represents the temperature anomalies over the NH during the same period as **T**_**Arc**._ The ordinary linear least squares solution determines parameters *a*_0_, *a*_1_, and *ε*. The AA index is the slope of the regression (*a*_1_ in Eq. (6)); thus, the AA index value depends on the change rate of the independent (**T**_**NH**_) and dependent (**T**_**Arc**_) variables over the regression interval. A previous study^27^ reported that this new regression-based AAI estimate is more stable than the estimate based on the ratio and can preserve the intrinsic mode of AA. In this study, the regression was operated with a 31-yr moving window (around a climatology baseline) to reconstruct the AAI during the past millennium. At this time scale, the AAI can be used to investigate the multidecadal variability in AA.

### Mann-Kendall trend detection

The Mann-Kendall method^57^ is a nonparametric test for monotonic trends. This method does not assume a specific distribution of the data and is insensitive to outliers. Because of these advantages, the Mann-Kendall method has been widely used in climatic trend analysis^58,59^. In order to limit the influence of autocorrelation of time series on the Mann-Kendall trend detection, we used a modified Mann-Kendall test^57^ which calculates an effective sample size by considering serial correlations. Based on the partial autocorrelation functions of the AA indices, the indices can be modeled as first-order autoregressive (AR(1)) processes; thus the modified Mann-Kendall tests were calculated based on the AR(1) assumption.

### Adjusted correlation *p*-values

To account for the fact that the AA index was calculated using 31 years moving windows, we calculated effective sample size in the correlation analysis by dividing the actual sample size by 30 and used this effective sample size in the calculation of the correlation *p*-values.

## Supplementary information

Supplementary Information

Description of Additional Supplementary Files

Supplementary Data 1

## Data Availability

All data used in this study are freely accessible. The proxies were selected from the PAGES2k Consortium (http://www.pastglobalchanges.org). CMIP5 simulations were downloaded from the Earth System Grid Federation (https://esgf.llnl.gov/). The Goosse2012 reconstruction, proxy-based reconstructions and transient simulations used in Fig.5 were downloaded from the NOAA Paleoclimatology Data Centre (https://www.ncdc.noaa.gov/data-access/paleoclimatology-data). The Steiger2018 reconstruction can be downloaded from 10.5281/zenodo.1154913. The PDA-based reconstruction of this study was released at the National Tibetan Plateau Data Centre (https://data.tpdc.ac.cn/en/disallow/201553d9-9b6a-4793-954c-7eff9e124959/). Other data used in this study are attached in Supplementary Data 1.

## Code availability

The PDA source codes^60^ of this study is available at 10.5281/zenodo.6086564. More codes in this study are available from the corresponding author upon request.
