# Supplementary material for: Arctic amplification modulated by Atlantic Multidecadal Oscillation and greenhouse forcing on multidecadal to century scales
Source: Nat Commun. 2022 Apr 6;13:1865. doi: 10.1038/s41467-022-29523-x (PMC8987036; doi:10.1038/s41467-022-29523-x)
Supplement: Supplementary file 1 — Supplementary Information [file 41467_2022_29523_MOESM1_ESM.pdf]

Supplementary Information for

Arctic amplification modulated by Atlantic Multidecadal Oscillation and greenhouse forcing on multidecadal to century scales

Miao Fang, Xin Li*, Hans W. Chen*, Deliang Chen

Correspondence to: xinli@itpcas.ac.cn and hans.chen@nateko.lu.se

This file includes:

Supplementary Methods and Text
Figs. S1 to S7
Table S1

Supplementary Methods

Measures of verification

The verification of the PDA-based reconstruction was based on two primary verification metrics, the (Pearson) correlation coefficient (*r*) and the coefficient of efficiency (CE). *r* is a measure of the strength and direction of the linear relationship between two variables. CE is a measure of the differences between the values of two variables. Given two time series **X** and **V** of the *N* values of two variables (e.g., **X** is the data to be verified and **V** is the reference dataset), the two measures are defined as follows:

$$r = \frac{1}{N}\sum_{i=1}^{n}\left(\frac{(\mathbf{X}_i - \bar{\mathbf{X}})(\mathbf{V}_i - \bar{\mathbf{V}})}{\sigma_x \sigma_v}\right) \quad \text{(S1)}$$

$$\text{CE} = 1 - \frac{\sum_{i=1}^{N}(\mathbf{V}_i - \mathbf{X}_i)^2}{\sum_{i=1}^{N}(\mathbf{V}_i - \bar{\mathbf{V}})^2} \quad \text{(S2)}$$

Here, an overbar represents a mean value, and σ is the standard deviation. The *r* ranges from -1 to 1. The CE can range from −∞ to 1. A CE that is close to 1 indicates a good match between the two data, while a CE that is close to 0 indicates that the **X** data are close to the average value of the reference data; that is, overall, the **X** data are credible. If CE is much less than 0, the **X** data are not credible. In the pseudo-proxy evaluation experiment, we set the CE values associated with the MPI-ESM-P simulation (i.e., the prior) as the basis for the pseudo-proxy evaluation. In this case, negative CE values associated with the PDA-based reconstruction still reflect improvement upon the model simulation, as long as the CE values associated with the PDA-based reconstruction are greater than the CE values associated with the model simulation at the same locations[1].

The correlation measures signal timing and is not affected by errors in signal amplitude or bias. CE is affected by these factors, and as such, it is a useful measure for these aspects of the reconstruction. The sensitivity of CE to biases depends on the definition of a reference time period to define anomalies, which may be nonoverlapping between proxies, calibration data, and prior data. A known issue with CE is that if the means over the verification period for the proxies and the verification data are different, then CE can be negative even when the reconstruction is skillful[1].

Supplementary Text

(1) Verification against observational datasets

The PDA reconstruction during the instrumental period (i.e., 1850-2000AD) was verified against observational temperature datasets in both the time domain and space field (Fig. S1). The observational temperature datasets used in the verification include HadCRUT4[2], Berkeley Earth Surface Temperatures (hereinafter BEST)[3], NASA GISS

Surface Temperature Analysis (hereinafter GISTEMP)[4], and NOAA/NCEI Land and Ocean Temperature v3.2.1 (hereinafter NCDC)[5], as well as the gridded temperature dataset of CRU TS v4.01 (hereinafter CRU)[6]. Two primary metrics are considered, i.e., the *r* and CE. In addition, two other temperature datasets, i.e., a historical simulation experiment (1850-2005 AD) of MPI-ESM-P in the CMIP5 historical project[7] and 20CR-V2c reanalysis[8] produced by online data assimilation using the weather forecast model and global pressure observations, were also verified against the above observations to further compare the quality of our PDA reconstruction with model simulation and assimilation results based on instrumental observations.

The verifications show that PDA-based temperature anomalies agree well with the observed anomalies, with correlation values of 0.72~0.79 (all correlations are statistically significant at the α=0.01 level) and CE values of 0.02~0.60. All datasets (including observations, simulation, reanalysis and reconstruction) show obvious warming trends from 1850-2000 AD. This agreement reflects the close trends among all the anomalies. Although GISTEMP data were used to calibrate the forward model (i.e., the proxy system model, PSM), the PDA reconstruction shows higher correlations and CEs with BEST, HadCRUT4 and NCDC than with GISTEMP. In addition, all observational series are completely contained in the uncertainty range (95% confidence interval) of the PDA reconstruction, which indicates that the estimate of the uncertainty interval of the PDA reconstruction is reasonable. Furthermore, although the MPI-ESM-P simulation exhibits high correlations with observations, i.e., from 0.70 to 0.79 (all the correlations are statistically significant at the α=0.01 level), the CE values associated with the MPI-ESP-P simulation (i.e., -3.64~0.07) are obviously lower than those associated with the PDA reconstruction and 20CR-V2c, which suggests that the model simulation contains a larger bias than the data assimilation results. This bias is likely the consequences of model "drift".

For the grid-to-grid verifications, more than 99% of the correlation values between 20CR-V2c and CRU TS v4.01 are positive and with a mean of 0.62; the performance of the PDA reconstruction is less than to that of 20CR-V2c, but nevertheless more than 95% of the correlation values are positive and have a mean of 0.35, which indicates that the PDA reconstruction agrees well with the observations in terms of the timing of the temperature variations. For the model simulation, the percentage of positive correlation values is 82%, and the mean is 0.16, which indicates that the model simulation quality is obviously lower than those of the former two datasets. For the spatial distributions of CE values, we can see that the PDA reconstruction has the highest CE value, followed by the 20CR-V2c reanalysis and lastly the MPI-ESM-P simulation. Grid-to-grid verifications suggest that the data assimilation results, whether assimilating instrumental data (i.e., 20CR-V2c) or assimilating natural proxies (i.e., PDA reconstruction), are always superior to model simulations in terms of correlation and bias.

Admittedly, the quality of the 20CR-V2c reanalysis is higher than that of the PDA reconstruction in terms of the correlation with the observations, which is not surprising

given the amount, spatial distribution and quality of the assimilated surface pressure observations in the 20CR-V2c compared with the proxy data in the PDA. However, notably, for the NH averaged anomalies, the difference between the 20CR-V2c reanalysis and PDA reconstruction is small in terms of the correlations. For the NH temperature field, although there is a relatively large difference between the two datasets in the metric correlation means (0.62 for 20CR-V2c and 0.35 for PDA), there is a small difference in the proportion of positive correlation values (99% for 20CR-V2c and 95% for PDA). Additionally, the CE mean associated with the PDA reconstruction (-0.13) is larger than that of the 20CR-V2c reanalysis (-0.27). Overall, this finding indicates that the reconstructed temperature field based on the PDA approach has relatively high reliability that is close to that of the 20CR-V2c reanalysis; hence, the PDA reconstruction can be used to analyze the NH temperature field patterns and other field-related issues.

(2) Verification against proxy-based reconstruction

In order to place the PDA-based reconstruction in the context of previous well-known reconstructions, we show the NH-averaged temperature anomalies during the last millennium reconstructed based on multi-types proxies and the PDA approach. From Fig. S2 we can see that the PDA-based temperature anomalies are in reasonable agreement with several well-known reconstructions. The correlations between the PDA-based reconstruction and several proxy-based reconstructions range from 0.47 to 0.74 with a mean of 0.61, and more than half of the proxy-based reconstructions correlate at 0.6 or higher with the PDA-based temperature anomalies. The abovementioned correlations are statistically significant at the $\alpha = 0.01$ level. In addition, as in the proxy-based reconstructions, the NH-averaged temperature of the PDA-based reconstruction shows a cooling trend over the preindustrial era and a sharp warming trend during the industrial era. Superimposed upon those two trends are multidecadal oscillations, including climatic features consistent with the Medieval Climate Anomaly and the Little Ice Age. Those consistencies reflect the close trends among all the reconstructions mentioned in Fig. S2, that is to say, the PDA-based reconstruction agrees well with previous proxy-based reconstructions. In addition, compared to the proxy-based reconstructions, the PDA-based reconstruction has the strong advantage of providing spatiotemporally continuous temperature fields, with consistent physics constrained by climate models.

(3) Pseudo-proxy experiment verifications

We conducted a pseudo-proxy experiment to assess the performance of the PDA-based reconstruction during the past millennium. In the pseudo-proxy experiment, 75% of the observational proxies were randomly sampled and assimilated (Fig. S3a), and the remaining 25% observational proxies were used to verify the PDA-based reconstruction (Fig. S3b).

We compared the 25% observational-based records with the estimates of the proxy values from the PDA-based reconstruction and MPI-ESM-P millennium simulation using a proxy system model (Equation (5)) over the past millennium. This type of comparison can directly indicate how close the simulated proxy is to the corresponding observational proxy and demonstrate the performances of the PDA-based reconstruction and MPI-ESM-P simulation. The pseudo-proxy experiment shows that the PDA-based reconstruction clearly improves the temperature estimates compared to the prior MPI-ESM-P simulation. Indeed, overall, the PDA-based reconstruction is more reliable in terms of the r (Fig. S3c, d) and CE than the MPI-ESM-P simulation (Fig. S3e, f).

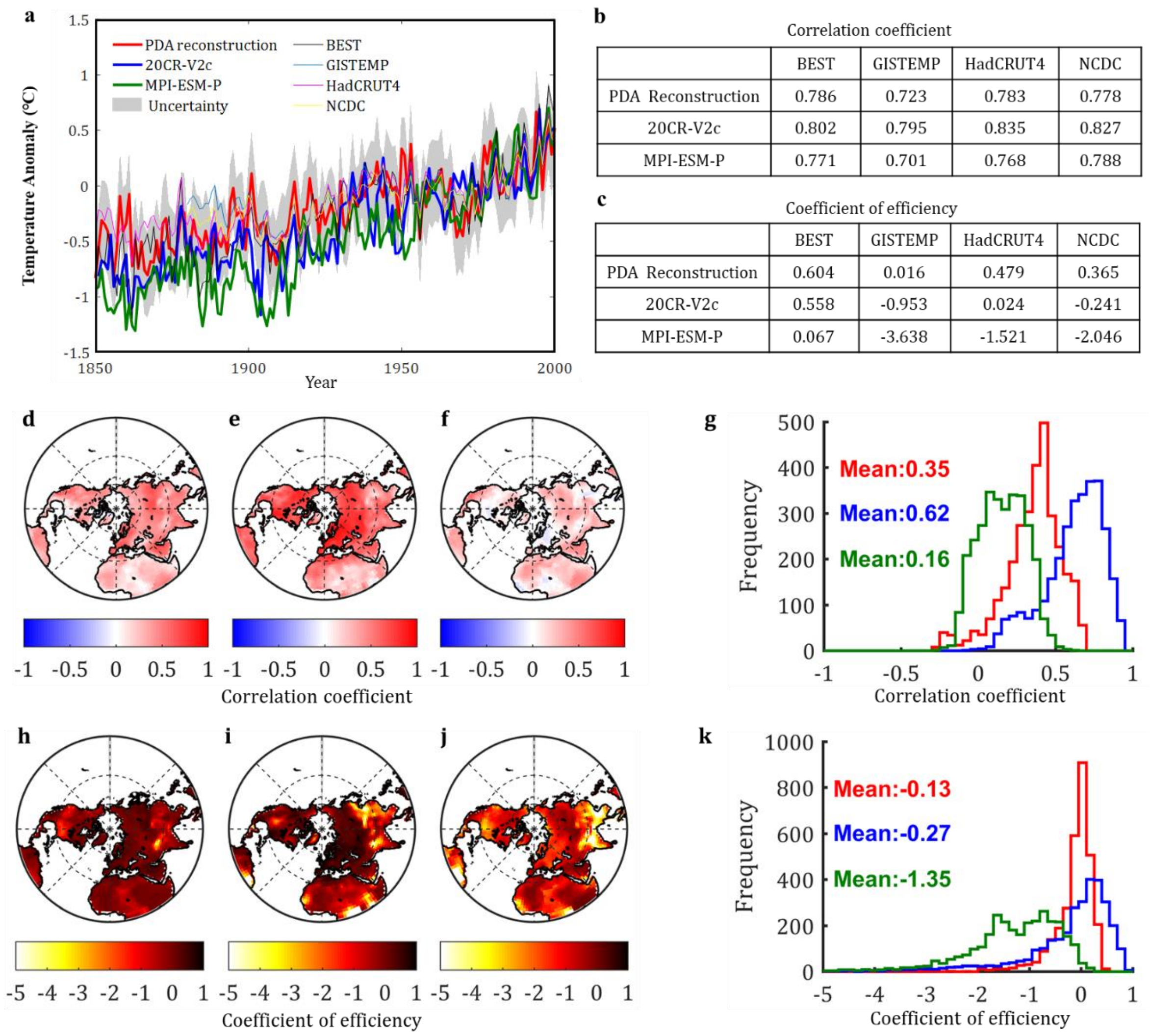

b Correlation coefficient

	BEST	GISTEMP	HadCRUT4	NCDC
PDA Reconstruction	0.786	0.723	0.783	0.778
20CR-V2c	0.802	0.795	0.835	0.827
MPI-ESM-P	0.771	0.701	0.768	0.788

c Coefficient of efficiency

	BEST	GISTEMP	HadCRUT4	NCDC
PDA Reconstruction	0.604	0.016	0.479	0.365
20CR-V2c	0.558	-0.953	0.024	-0.241
MPI-ESM-P	0.067	-3.638	-1.521	-2.046

Fig. S1. NH averaged annual temperature anomalies (relative to the mean of 1961-1990 AD) during 1850-2000 AD and grid-to-grid verification of the PDA reconstruction, 20CR-V2c reanalysis and MPI-ESM-P climate simulation against observations in terms of correlation coefficient and coefficient of efficiency. (**a**) Observational datasets (thin lines), PDA reconstruction (thick red line), 20CR-V2c reanalysis (thick blue line) and MPI-ESM-P simulation (thick green line). The grey band denotes the uncertainty range of PDA reconstruction (95% confidence interval). All anomalies are relative to the mean value from 1961-1990 AD. (**b**) Correlations and (**c**) coefficients of efficiency between the PDA reconstruction, MPI-ESM-P simulation and 20CR-V2c reanalysis and observations from 1850-2000 AD. All of the correlation coefficients are statistically significant at the α=0.01 level. (**d**), (**e**) and (**f**) The spatial patterns of the correlations between gridded CRU temperature and PDA reconstruction, 20CR-V2c reanalysis and MPI-ESM-P simulation from 1901-2000 AD. **(g)** The histograms of correlation values corresponding to the left three correlation coefficient fields (red line for PDA reconstruction, blue line for 20CR-V2c reanalysis, green line for the MPI-ESM-P simulation). All the correlations are statistically significant at the α=0.01 level. (**h**), (**i**) and (**j**) The spatial patterns of coefficients of efficiency between gridded CRU temperature and PDA reconstruction, 20CR-V2c reanalysis and MPI-ESM-P simulation from 1901-2000 AD, respectively. (**k**) The histograms of coefficient of efficiency values corresponding to the left three coefficient of efficiency fields (red line for PDA reconstruction, blue line for 20CR-V2c reanalysis, green line for MPI-ESM-P simulation). The correlation coefficient and coefficient of efficiency values were computed based on the annual temperature anomaly without any smoothing.

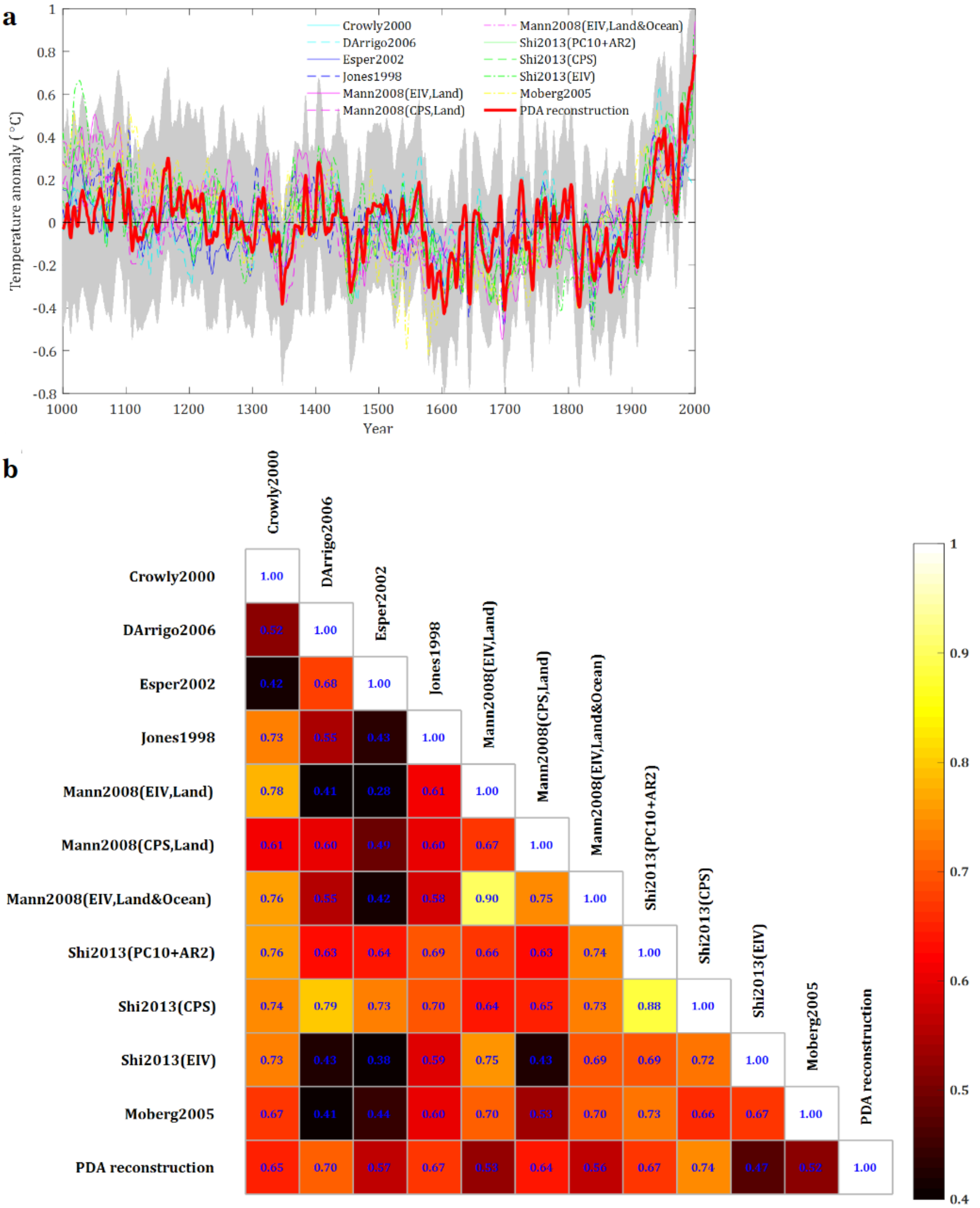

Fig. S2. The PDA reconstruction and previous proxy-based reconstructions. (**a**) Reconstructed annual temperature anomalies based on the PDA approach and proxy-based approach. Proxy-based reconstructions include Jones1998[9], Crowley2000[10], Esper2002[11], Moberg2005[12], DArrigo2006[13], Mann2008[14] and Shi2013[15]. All the time series are anomalous relative to their individual millennial means. The PDA reconstruction was filtered by the loess method (span = 0.01), while other proxy-based reconstructions retained their original series without post-smoothing. (**b**) Verification of NH averaged annual temperature anomalies over the past millennium (1000-1979 AD) in terms of the correlation (because the reconstruction of Moberg2005 ends in 1979 AD, the correlations were accordingly computed based on temperature anomalies from 1000-1979 AD). All the time series are anomalous relative to their individual millennial means, and their original series are retrained without post-smoothing. All the correlation coefficients are statistically significant at the α=0.01 level.

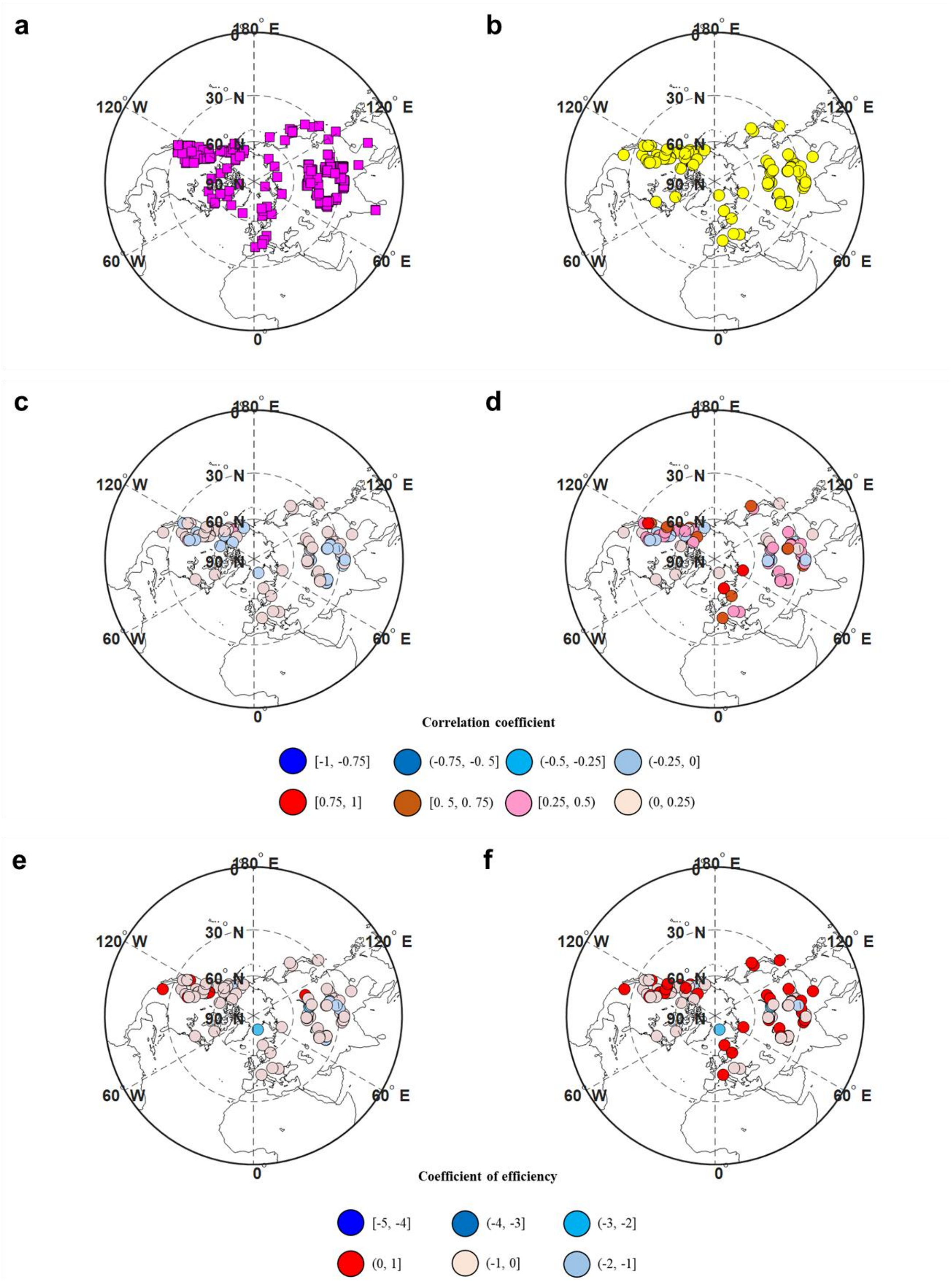

Fig. S3. The spatial distributions of observational proxies in pseudo-proxy verifications and the spatial distributions of correlation coefficient and coefficient of efficiency values in the pseudo-proxy experiment. (**a**) 75% of the observational proxies that were assimilated in the PDA process; (**b**) the remaining 25% of the observational proxies that were used to verify the PDA-based reconstruction. (**c**) Correlation coefficient values between the remaining 25% observational-based records and the estimates of the proxy values from the MPI-ESM-P millennium simulation; (**d**) correlation coefficient values between the remaining 25% observational-based records and the estimates of the proxy values from the PDA-based reconstruction. (**e**) Coefficient of efficiency values between the remaining 25% observational-based records and the estimates of the proxy values from the MPI-ESM-P millennium simulation; (**f**) coefficient of efficiency values between the remaining 25% observational-based records and the estimates of the proxy values from the PDA-based reconstruction.

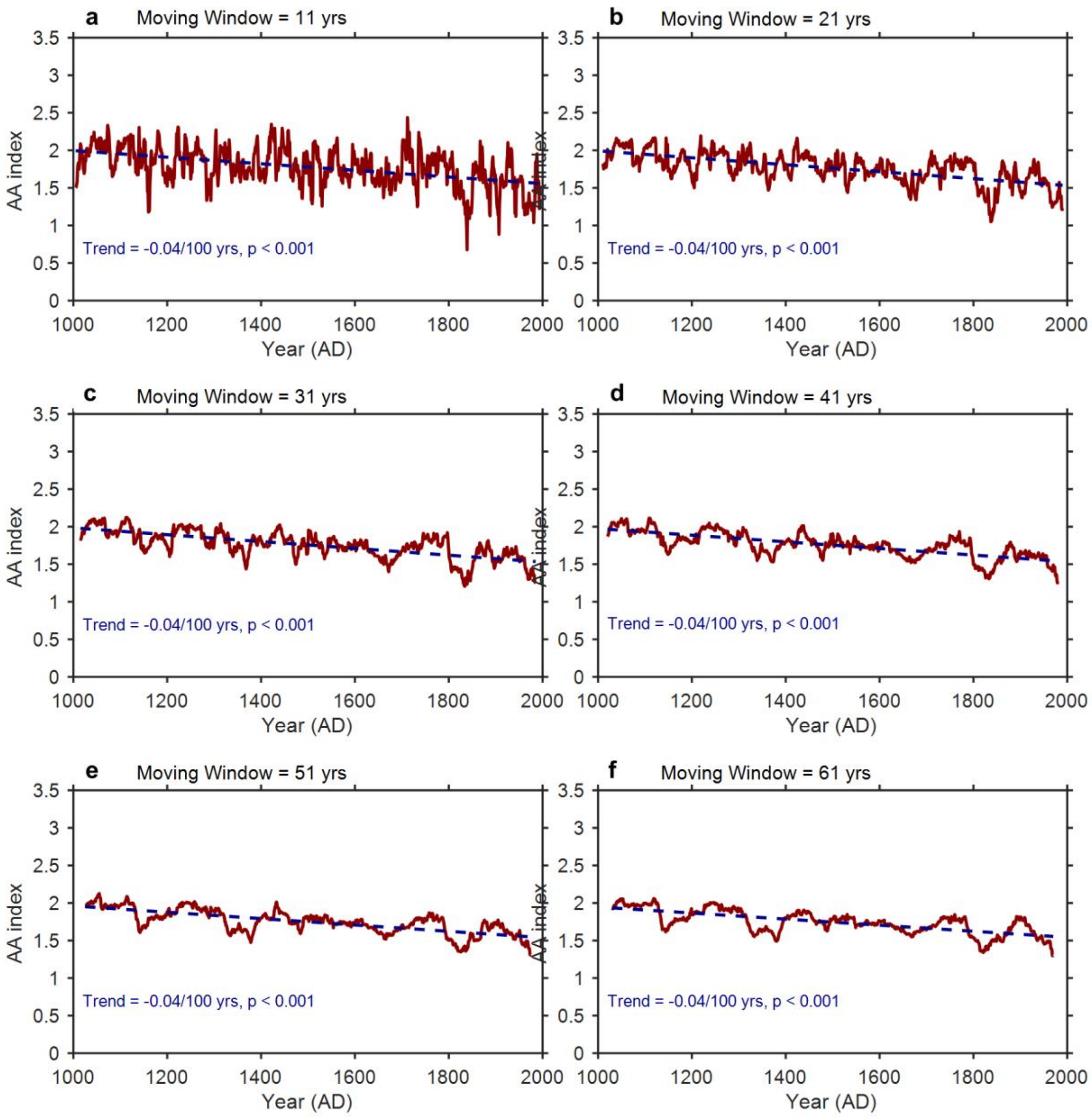

Fig. S4. Sensitive analysis of the reconstructed AA indices to the moving window size for the regression. The size of the time window was set to (**a**) 11 years, (**b**) 21 years, (**c**) 31 years, (**d**) 41 years, (**e**) 51 years, and (**f**) 61 years.

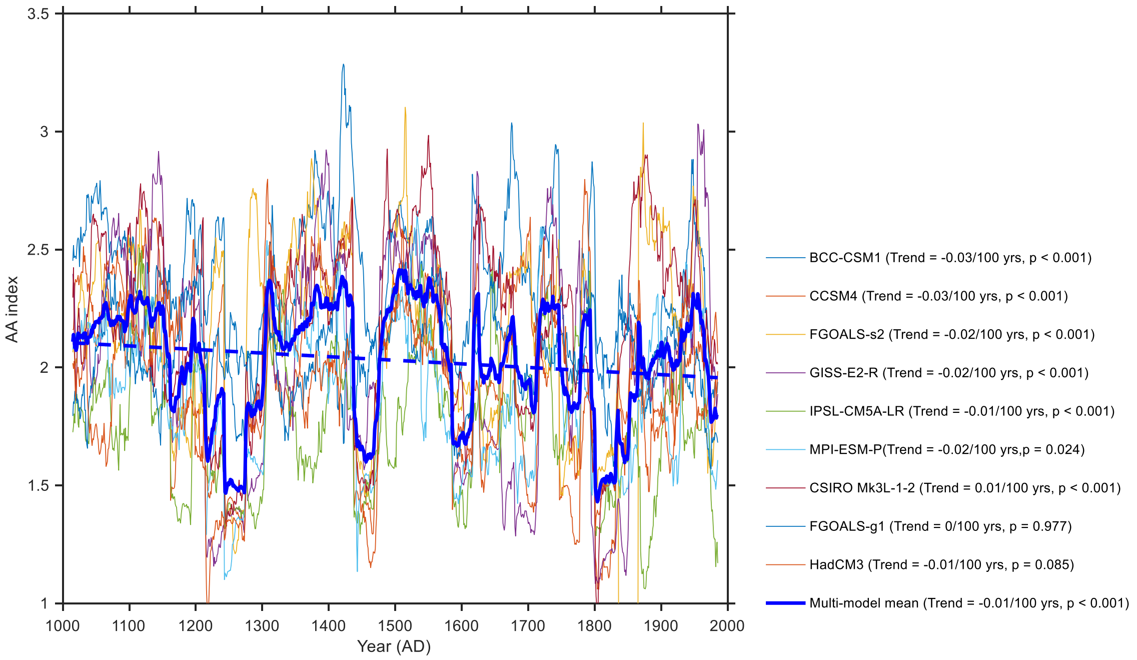

Fig. S5. The reconstructed AA indices and estimated AA trends during the past millennia derived from the CMIP5 multi-model simulations. The multi-model ensemble mean AA index was calculated by computing the AA index from each member first and then averaging over the AA indices from the ensemble members. The blue dotted line is the long-term trend of the multi-model ensemble mean AA index time series, which was computed via Mann-Kendall trend detection (see Methods).

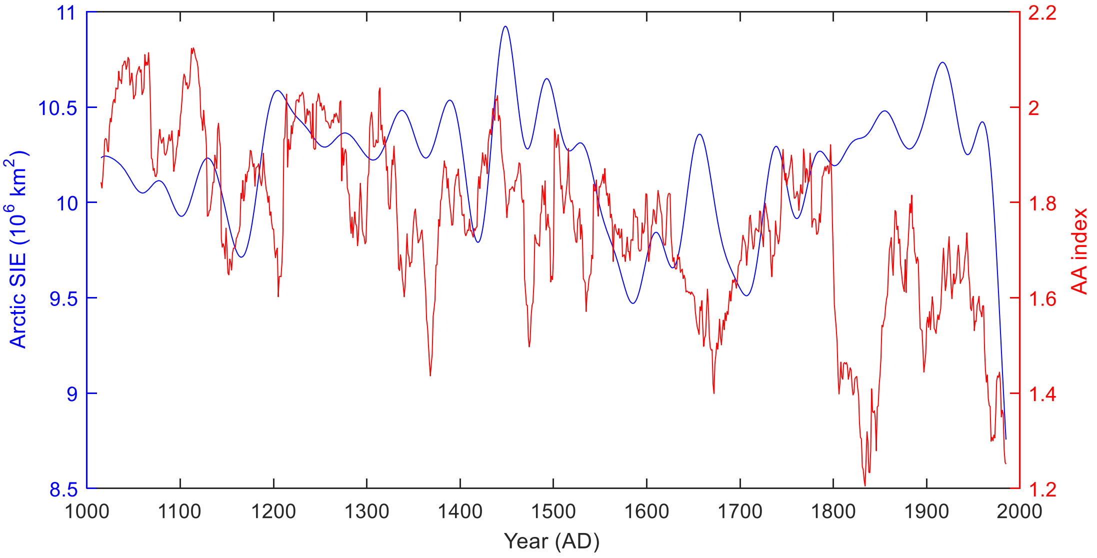

Fig. S6. The reconstructed Arctic sea ice extent (SIE) time series[16] and the AA index in this study.

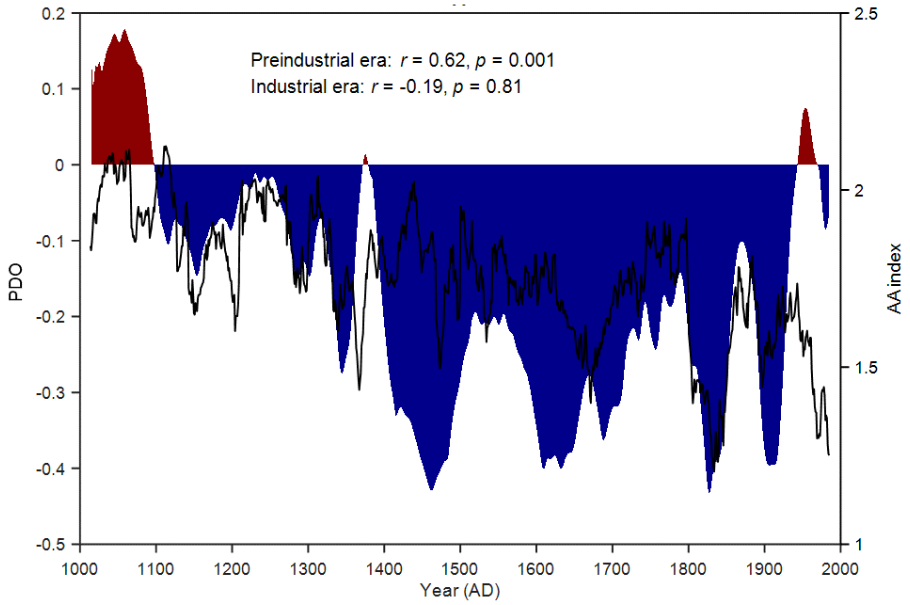

Fig. S7. Co-variation between the reconstructed millennial AA index and PDO modes. The reconstructed millennial AA index (black line) derived from the PDA-based reconstruction and the reconstructed PDO index (shading) based on multi-type proxies[17].

Table S1. Overview of paleoclimate temperature reconstructions used in this study.

	Goosse2012	Steiger2018	This study
**Climate model**	LOVECLIM[18]	CESM[19]	MPI-ESM-P[20]
**Data assimilation algorithm**	Particle filter[21]	EnSRF[22]	EnSRF[22]
**Proxy dataset**	Mann2009[17]	PAGES2k Consortium[23] and additional tree ring proxy[24]	PAGES2k Consortium[23]
**Ensemble size**	96	998	500
**Spatial coverage**	Global	Global	Northern Hemisphere

Supplementary References

1. Hakim, G. J. et al. The last millennium climate reanalysis project: framework and first results. *J. Geophys. Res. Atmos.* **121**, 6745-6764 (2016).
2. Morice, C. P., Kennedy, J. J., Rayner, N. A. & Jones, P. D. Quantifying uncertainties in global and regional temperature change using an ensemble of observational estimates: the HadCRUT4 data set. *J. Geophys. Res. Atmos*. **117**, D08101 (2012).
3. Rohde, R., Muller, R., Jacobsen, R., Perlmutter, S. & Mosher, S. Berkeley earth temperature averaging process. *Geoinfor. Geostat. An Overview* **1**, 1-13 (2013).
4. Hansen, J., Ruedy, R., Sato, M. & Lo, K. Global surface temperature change. *Rev. Geophys.* **48**, RG4004 (2010).
5. Smith, T. M., Reynolds, R. W., Peterson, T. & Lawrimore, C. J. Improvements to NOAA's historical merged land-ocean surface temperature analysis (1880-2006). *J. Clim.* **21**, 2283-2296 (2008).
6. Harris, I. P., Jones, P. D., Osborn, T. J. & Lister, D. H. Updated high-resolution grids of monthly climatic observations-the CRU TS3.10 dataset. *Int. J. Climatol.* **34**, 623-642 (2014).
7. Taylor, K. E., Stouffer, R. J. & Meehl, G. A. An overview of CMIP5 and the experiment design. *Bull. Am. Meteorol. Soc.* **93**, 485-498 (2012).
8. Compo, G. P. et al. The twentieth century reanalysis project. *Q. J. R. Meteorol. Soc.* **137**, 1-28 (2011).
9. Jones, P. D., Briffa, K. R., Barnett, T. P. & Tett, S. F. B. High-resolution paleoclimatic records for the last millennium: Interpretation, integration and comparison with general circulation model control-run temperatures. *Holocene* **8**, 455-471 (1998).
10. Crowley, J. T. Causes of climate change over the past 1000 years. *Science* **289**, 270-277 (2000).
11. Esper, J., Cook, E. R. & Schweingruber, F. H. Low-frequency signals in long tree-ring chronologies for reconstructing past temperature variability. *Science* **295**, 2250-2253 (2002).
12. Moberg, A. et al. Highly variable Northern Hemisphere temperatures reconstructed from low- and high-resolution proxy data. *Nature* **433**, 613-617 (2005).
13. D'Arrigo, R., Wilson, R. & Jacoby, G. On the long-term context for late twentieth century warming. *J. Geophys. Res. Atmos.* **111**, D03103 (2006).
14. Mann, M. E. et al. Proxy-based reconstructions of hemispheric and global surface temperature variations over the past two millennia. *Proc. Natl. Acad. Sci. U.S.A.* **105**, 13252-13257 (2008).
15. Shi, F. et al. Northern hemisphere temperature reconstruction during the last millennium using multiple annual proxies. *Clim. Res.* **56**, 231-244 (2013).
16. Kinnard, C. et al. Reconstructed changes in arctic sea ice over the past 1,450 years. *Nature* **479**, 509-512 (2011).
17. Mann, M., Zhang, Z. & Rutherford, S. Global signatures and dynamical origins of the Little Ice Age and Medieval Climate Anomaly. *Science* **326**, 1256-1260 (2009).

18. Goosse, H. et al. Description of the earth system model of intermediate complexity LOVECLIM version 1.2. *Geosci. Model Dev*. **3**, 603-633 (2010).

19. Otto-Bliesner, B. L. et al. Climate variability and change since 850 CE: An ensemble approach with the community earth system model. *B. Am. Meteorol. Soc*. **97**, 735-754 (2016).

20. Jungclaus, J. H. et al. Characteristics of the ocean simulations in the Max Planck Institute Ocean Model (MPIOM) the ocean component of the MPI-Earth system model. *J. Adv. Model Earth Sy*. **5**, 422-446 (2013).

21. van Leeuwen, P. J. Particle filtering in geophysical systems. *Mon. Weather Rev*. **137**, 4089-4114 (2009).

22. Steiger, N. J., Hakim, G. J., Steig, E. J., Battisti, D. S. & Roe, G. H. Assimilation of time-averaged pseudoproxies for climate reconstruction. *J. Clim*. **27**, 426-441 (2014).

23. PAGES2k Consortium. A global multiproxy database for temperature reconstructions of the common era. *Sci. Data* **4**, 170088 (2017).

24. Breitenmoser, P., Brönnimann, S. & Frank, D. Forward modelling of tree-ring width and comparison with a global network of tree-ring chronologies. *Clim. Past* **10**, 437-449 (2014).
