# Supplementary material for: Arctic amplification modulated by Atlantic Multidecadal Oscillation and greenhouse forcing on multidecadal to century scales
Source: Nat Commun. 2022 Apr 6;13:1865. doi: 10.1038/s41467-022-29523-x (PMC8987036; doi:10.1038/s41467-022-29523-x)
Supplement: Supplementary file 2 — Description of Additional Supplementary Files [file 41467_2022_29523_MOESM2_ESM.pdf]

Description of Additional Supplementary Files

File Name: Supplementary Data 1
Description: It contains the forcings and internal modes of climate system and the millennial AA indices.
